# Job Satisfaction in Nursing Practice: A Descriptive and Comparative Study Across Organizational and Professional Groups

**DOI:** 10.3390/nursrep16050164

**Published:** 2026-05-13

**Authors:** Olinda Monsanto, António Nunes, Ana João

**Affiliations:** 1 Local Health Unit of Castelo Branco, Specialty Services 1, 6000-406 Castelo Branco, Portugal; 2 NECE: Research Centre for Business Sciences, Department of Management and Economics, University of Beira Interior, 6201-001 Covilhã, Portugal; anunes@ubi.pt; 3 Comprehensive Health Research Center (CHRC), School of Health, Polytechnic Institute of Santarém, 2001-904 Santarém, Portugal; alsjoao@uevora.pt

**Keywords:** job satisfaction, sociodemographic factors, workplace, nursing, health personnel

## Abstract

**Background:** Nurses’ job satisfaction is an important factor associated with motivation, retention, and performance, potentially influencing the quality and safety of healthcare delivery. Identifying organizational and professional determinants of job satisfaction is essential for the sustainability of healthcare systems. **Objective:** This study aims to describe nurses’ job satisfaction across its multiple dimensions and examine differences in job satisfaction dimensions across sociodemographic and professional groups. **Methods:** A quantitative, cross-sectional, descriptive–correlational study was conducted with 153 nurses. Data were collected between October and December 2024 using an online questionnaire, with a response rate of 28.9%, which included the Escala de Satisfação dos Enfermeiros com o Trabalho (ESET). Descriptive and inferential statistical analyses were performed, with the significance level set at 0.05. **Results:** Moderate levels of job satisfaction predominated among participants (75.8%), with 5.2% of participants reporting low satisfaction. The highest mean scores were observed in satisfaction with co-workers and professional recognition, while the lowest scores were found in the recognition and remuneration dimension. Statistically significant differences in mean job satisfaction scores were observed across groups defined by variables such as work setting, work schedule, weekly workload, and employment across multiple workplaces. **Conclusions:** Nurses’ job satisfaction is multidimensional and varies across different professional and organizational groups. These findings highlight areas of lower job satisfaction that may represent priorities for future organizational assessment and management attention.

## 1. Introduction

Job satisfaction is a fundamental component of the effective functioning of healthcare organizations, influencing nurses’ motivation, the quality of care provided, and workforce retention. Nurses’ job satisfaction has a direct impact on the quality of healthcare services and patient satisfaction [1].

Job satisfaction has been defined as a positive emotional state resulting from the individual’s evaluation of their work, organizational conditions, interpersonal relationships, and opportunities for development [2,3,4,5]. It is widely described as a complex and multifaceted phenomenon, shaped by specific organizational, environmental, and personal contexts, which is reflected in attitudes and emotional responses toward work [6].

Job satisfaction results from the interaction between employees’ expectations and the conditions provided by the organization, being essential for institutional success, growth, and the personal fulfilment of workers [3]. In this context, satisfaction emerges from the alignment between professional expectations and the actual conditions offered by the organization and constitutes a central element in healthcare human resource management.

In the field of nursing, job satisfaction assumes particular relevance due to the emotional and technical complexity of the profession, the high level of clinical responsibility, and the direct impact of care on patient safety and well-being. In addition to reflecting the organizational climate, nurses’ satisfaction directly influences institutional performance, while dissatisfaction may compromise service quality and patient perceptions [6].

Recent studies indicate that factors such as workload, physical working conditions, recognition, autonomy, relationships with colleagues and management, and opportunities for career progression are critical determinants of nurses’ job satisfaction [2,7,8,9].

The literature also highlights that low levels of job satisfaction may compromise the quality of care, increase absenteeism, and accelerate the intention to leave the profession [1].

The Portuguese Order of Nurses has consistently emphasized that the organizational environment, opportunities for professional development and career progression, and professional recognition are critical factors for nurses’ satisfaction and retention [4,10].

Local Health Units, by integrating primary and hospital care within a single organizational model, encompass distinct work settings with different care demands, leadership models, and work dynamics. This heterogeneity may significantly influence nurses’ professional experiences and levels of job satisfaction, making its analysis particularly relevant within an integrated organizational context.

Understanding job satisfaction levels and their association with sociodemographic and professional characteristics allows for the identification of priority areas for intervention, supports evidence-based management decisions, and guides human resource policies aimed at promoting healthy and sustainable work environments. Beyond its impact on workforce retention, these factors may also affect the quality of care provided and patient safety.

Within this framework, the present study aims to (i) describe nurses’ job satisfaction across its multiple dimensions and (ii) explore the associations between job satisfaction dimensions and sociodemographic and professional variables.

## 2. Materials and Methods

### 2.1. Study Design

This study is a non-experimental investigation with a quantitative approach, adopting a descriptive, cross-sectional, comparative, and correlational design, involving Portuguese nurses from a Local Health Unit.

The study followed a cross-sectional design, with data collected at a single point in time, allowing the capture of a specific context within a defined period, often described as a “snapshot” of a specific moment [11]. This design enabled the analysis of the prevalence of nurses’ job satisfaction at a particular moment.

No variables were manipulated, as the focus was on analyzing the relationships between sociodemographic/professional characteristics and levels of job satisfaction.

### 2.2. Population and Sample

In this study, the target population consisted of nurses, with the sample comprising professionals from the Local Health Unit of Castelo Branco (LHUCB) who agreed to participate.

The LHUCB is a public enterprise entity established on 1 January 2010, resulting from the merger of Hospital Amato Lusitano with the Primary Healthcare Centre Groups of Beira Interior Sul and Pinhal Interior Sul, in the district of Castelo Branco. Its mission is to provide integrated primary, hospital, and continuing care, ensuring quality, equity, and professional training. The structure includes a reference hospital with a medical-surgical emergency department and approximately 205 beds, as well as nine primary healthcare centers (eight Personalized Healthcare Units and three Family Health Units) organized into two Primary Healthcare Centre Groups, ensuring comprehensive and accessible healthcare coverage [12].

Data were collected over a two-month period, between October and December 2024, using an online questionnaire administered via the Google Forms platform. The target population consisted of nurses, and the sample comprised professionals from the Local Health Unit of Castelo Branco (LHUCB) who voluntarily agreed to participate.

The questionnaire was distributed via institutional email to 550 nurses from the LHUCB. Of these, 20 emails were not successfully delivered. A total of 153 valid responses were obtained, corresponding to a response rate of 28.9% and constituting the final study sample.

A non-probabilistic convenience sampling strategy was adopted, as participants were selected based on their accessibility and willingness to participate. Additionally, and as a complementary approach, participants were encouraged to share the questionnaire link with other professionals who met the inclusion criteria, with the aim of increasing the response rate and enhancing sample heterogeneity [13]. However, the recruitment process relied primarily on the initial institutional distribution, maintaining the voluntary, anonymous, and uncontrolled nature of participation.

The inclusion criteria comprised (i) registered nurses working in the Local Health Unit of Castelo Branco, either in hospital or primary healthcare settings; (ii) active professional practice during the data collection period; and (iii) voluntary agreement to participate in the study.

Exclusion criteria included (i) professionals not performing clinical functions at the time of data collection (e.g., on leave or in exclusively administrative roles); and (ii) incomplete questionnaire responses.

The final sample included 153 nurses, with a mean age of 45.73 years (standard deviation = 9.77). The sample was predominantly female (82.4%) and composed mainly of professionals with more than 20 years of experience.

### 2.3. Data Collection Instrument

Data were collected through an online questionnaire administered via Google Forms. The questionnaire included a brief explanation of the study’s purpose, ensuring confidentiality and anonymity.

The Escala de Satisfação dos Enfermeiros com o Trabalho (ESET) was used to assess nurses’ job satisfaction in Portugal. This scale was developed and validated by João et al. [14]. It was designed to evaluate nurses’ satisfaction in hospital settings, aligning with the objectives of the present study.

The ESET consists of 37 items distributed across six dimensions:

- Satisfaction with Leadership (items 5, 6, 10, 14, 17, 19, 21, 24, 28, 29, 33, and 35).
- Satisfaction with Organization and Resources (items 12, 13, 18, 22, 23, 25, 26, and 30).
- Satisfaction with Professional Recognition (items 15, 16, 34, 36, and 37).
- Satisfaction with Co-workers (items 1, 2, 4, 9, and 20).
- Satisfaction with Recognition and Remuneration (items 3, 8, 27, 31, and 32).
- Satisfaction with Staffing Levels (items 7 and 11).

Each item is rated on a five-point Likert scale. The total score is obtained by summing the values of the 37 items. Scores are interpreted as follows: 1–61 indicates low satisfaction; 62–123 indicates moderate satisfaction; and scores above 124 indicate high satisfaction.

The scale demonstrated high internal consistency in this sample (α > 0.80).

### 2.4. Ethical Procedures

The study commenced after formal authorization from the healthcare institution and approval by the Ethics Committee of the Local Health Unit of Castelo Branco, issued on 27 September 2024. Throughout the study, the fundamental principles of bioethics—autonomy, non-maleficence, beneficence, and justice—were respected.

Authorization for the use of the ESET scale was requested and obtained from its author.

During the administration of the questionnaire, informed consent was obtained from all participants, ensuring anonymity, data confidentiality, and exclusive use for scientific purposes.

The questionnaire was designed to avoid any discomfort. Participants were informed that they would not receive any financial compensation and could withdraw at any time without consequences. Data were analyzed in aggregate form, securely stored, and deleted one month after the completion of the study, with access restricted exclusively to the research team.

### 2.5. Statistical Analysis

Data analysis was conducted in two stages: descriptive and inferential statistics. In the first stage, descriptive statistics were used to characterize the sociodemographic and professional profile of the sample, as well as to describe nurses’ job satisfaction levels. Absolute and relative frequencies were calculated for categorical variables, while measures of central tendency and dispersion (mean, standard deviation, minimum and maximum values) were computed for quantitative variables, allowing for a detailed characterization of data distribution.

Given the exploratory nature of the study, analyses were conducted across multiple dimensions of job satisfaction without predefined directional hypotheses.

Missing data were assessed prior to analysis. Cases with missing values were handled using listwise deletion. This approach was considered appropriate given the low proportion of missing data and the absence of systematic patterns in missingness.

In the second stage, inferential analysis was performed to examine differences in job satisfaction dimensions across sociodemographic and professional groups. Prior to inferential analysis, the assumptions for parametric testing were assessed. Normality of the distributions was evaluated using the Shapiro–Wilk test and inspection of Q–Q plots, while homogeneity of variances was assessed using Levene’s test.

Parametric tests were applied whenever the assumptions of normality and homogeneity of variances were met. The Student’s *t*-test was used to compare mean scores of job satisfaction dimensions between two independent groups (e.g., dichotomous sociodemographic or professional variables).

For comparisons involving three or more groups, one-way analysis of variance (ANOVA) was conducted, with the dimensions of job satisfaction as dependent variables and sociodemographic or professional characteristics as independent factors. When the assumption of homogeneity of variances was violated, Welch’s ANOVA was applied as a robust alternative.

When statistically significant differences were identified, post hoc comparisons were performed using both the least significant difference (LSD) test and the Bonferroni correction. The LSD test was used as a more sensitive exploratory approach, whereas Bonferroni-adjusted results were considered as a more conservative basis for interpretation, reducing the risk of type I error associated with multiple pairwise comparisons.

However, given the large number of statistical tests conducted across multiple ESET dimensions and sociodemographic and professional variables, no global correction for multiple comparisons was applied. Therefore, the analyses should be interpreted as exploratory, and statistically significant findings should be considered hypothesis-generating.

When the assumptions for parametric tests were not met, appropriate non-parametric alternatives (Mann–Whitney U test or Kruskal–Wallis test) were used.

A statistical significance level of α ≤ 0.05 was adopted.

Data processing was performed using Microsoft Excel for Microsoft 365 (Microsoft Corporation, Redmond, WA, USA). Statistical analyses were conducted using IBM SPSS Statistics for Windows, version 29.0 (IBM Corp., Armonk, NY, USA).

The internal consistency of the ESET was assessed using Cronbach’s alpha coefficient. The total scale demonstrated excellent reliability (α = 0.964), while the values for the individual dimensions ranged from 0.772 to 0.944, indicating adequate to very high internal consistency (Table 1).

## 3. Results

According to Table 2, the sample was predominantly female (82.4%), with males representing 16.3%, and 1.3% opting not to respond. Participants’ ages ranged from 26 to 64 years, with a mean of 45.73 years (SD = 9.77). The most represented age groups were 40–50 years (34.0%) and 50–60 years (28.8%).

Regarding marital status, most participants were married (58.8%), followed by those in a cohabiting relationship (17.6%), single (13.1%), divorced/separated (9.8%), and widowed (0.7%). Most participants reported having children (76.5%).

In terms of professional context, 77.8% of participants worked in hospital settings, 20.3% in primary healthcare, and 2.0% in other settings. Most participants worked in a single workplace (75.2%).

Concerning working conditions, 77.8% of participants worked between 35 and 40 h per week, 14.4% worked more than 40 h, and 7.8% worked fewer than 35 h. Regarding professional experience, the majority had more than 20 years of experience (59.5%), while only 6.5% had between 1 and 5 years of practice.

With regard to work schedules at the main workplace, 60.8% of participants worked in shifts, while 35.3% had a fixed schedule.

Table 3 presents the distribution of the sample according to the intention to change department or leave the current workplace. A total of 30.1% of participants reported an intention to change department or leave their current workplace, and 60.8% had considered leaving the profession at some point during their careers.

Analysis of Table 4 indicates that most participants reported moderate job satisfaction (n = 116, 75.8%), while 5.2% (n = 8) of the sample reported low satisfaction. Overall, 75.8% of nurses presented moderate levels of satisfaction, 19.0% high satisfaction, and 5.2% low satisfaction, with significant variation observed across the different dimensions of job satisfaction.

According to Table 5, participants reported higher levels of satisfaction with the quality of care provided (mean = 3.50) and patients’ respect (mean = 3.12). However, lower levels of satisfaction were observed regarding opportunities for career progression (mean = 1.45) and salary (mean = 1.58).

According to Table 6, at the level of the ESET scale dimensions, the highest mean levels of satisfaction were observed in “satisfaction with co-workers” (mean = 2.97) and “satisfaction with professional recognition” (mean = 2.94). However, the lowest level of job satisfaction was found in the dimension “satisfaction with recognition and remuneration” (mean = 1.82).

### Differences in Job Satisfaction Across Sociodemographic and Professional Variables

In the present study, analysis of Table 7 revealed no statistically significant differences across any dimensions of the job satisfaction scale when considering gender and age. Effect sizes were small across all dimensions (Cohen’s d ranging from −0.255 to 0.281; η^2^ ranging from 0.002 to 0.046), reinforcing the absence of meaningful differences between groups.

Regarding marital status, Welch’s ANOVA indicated significant differences in “satisfaction with co-workers” (F(3; 2.11) = 3.31, *p* = 0.022, η^2^ = 0.063) and “satisfaction with recognition and remuneration” (F(3; 1.42) = 3.68, *p* = 0.014, η^2^ = 0.069), indicating moderate effect sizes. Post hoc comparisons were conducted using both the LSD test and the Bonferroni correction. The LSD test suggested that divorced/separated and cohabiting nurses reported lower satisfaction with recognition and remuneration compared to married nurses (1.56 and 1.59 vs. 1.96). However, when applying the Bonferroni correction, only a limited number of pairwise differences remained statistically significant, highlighting the more conservative nature of this procedure.

Specifically, statistically significant differences were observed in satisfaction with co-workers between single and divorced/separated individuals (*p* = 0.029), and in satisfaction with recognition and remuneration between married individuals and those in a cohabiting relationship (*p* = 0.042). Across the remaining dimensions, no statistically significant differences were identified after Bonferroni adjustment (*p* > 0.05), indicating that differences between marital status groups were not consistently observed.

The variables having children and educational level/training did not show statistically significant differences across any ESET dimensions, with small effect sizes observed (Cohen’s d ranging from −0.259 to 0.306; η^2^ ranging from 0.005 to 0.034).

Concerning the professional setting, statistically significant differences were identified in the dimensions “satisfaction with professional recognition” (t(70.955) = −2.608, *p* = 0.006, d = −0.413), “satisfaction with recognition and remuneration” (t(41.077) = −3.429, *p* = 0.002, d = −0.691), and “satisfaction with staffing levels” (t(148) = −1.769, *p* = 0.039, d = −0.357). These results indicate small to moderate effect sizes, with the strongest effect observed in recognition and remuneration.

The variable “working in more than one workplace” showed statistically significant differences in “satisfaction with leadership” (t(151) = −1.858, *p* = 0.033, d = −0.348), “satisfaction with organization and resources” (t(151) = −2.063, *p* = 0.020, d = −0.386), “satisfaction with recognition and remuneration” (t(89.002) = −3.651, *p* = 0.001, d = −0.577), and “satisfaction with staffing levels” (t(151) = −2.280, *p* = 0.016, d = −0.427), reflecting predominantly moderate effect sizes.

Weekly working hours showed statistically significant differences only in the dimension of satisfaction with recognition and remuneration (F(2, 26.113) = 5.994, *p* = 0.007, η^2^ = 0.036), indicating a small effect size. However, when post hoc comparisons were examined using the Bonferroni correction, no statistically significant differences were identified between groups across any of the satisfaction dimensions (*p* > 0.05 for all comparisons).

Although a marginal difference was observed in satisfaction with remuneration valuation, particularly between individuals working 35–40 h and those working more than 40 h (*p* = 0.056), this result did not reach the adjusted threshold for statistical significance.

Regarding years of professional experience, no statistically significant differences were found, with consistently small effect sizes (η^2^ ranging from 0.012 to 0.051).

In relation to work schedule, statistically significant differences were observed in “satisfaction with leadership” (t(143) = 3.348, *p* = 0.001, d = 0.609), “satisfaction with organization and resources” (t(143) = 1.928, *p* = 0.028, d = 0.367), “satisfaction with professional recognition” (t(143) = 3.169, *p* = 0.001, d = 0.580), “satisfaction with recognition and remuneration” (t(93.406) = 3.833, *p* = 0.001, d = 0.664), and “satisfaction with staffing levels” (t(143) = 2.138, *p* = 0.034, d = 0.362). These results indicate moderate effect sizes, particularly for leadership and recognition-related dimensions.

Finally, the employment contract variable did not show statistically significant differences, with small effect sizes observed across all dimensions (Cohen’s d ranging from −0.318 to 0.109).

## 4. Discussion

Given the univariate nature of the analyses, the findings should be interpreted as exploratory differences between groups rather than evidence of independent associations. It was found that the majority of nurses (75.8%) reported moderate levels of job satisfaction, with only 19.0% indicating high satisfaction and 5.2% low satisfaction. These findings are consistent with previous studies [2,7,15].

A detailed analysis of the satisfaction dimensions revealed an interesting pattern: the highest mean scores were observed in satisfaction with co-workers (2.97) and professional recognition (2.94), whereas satisfaction with recognition and remuneration presented the lowest mean score (1.82). These findings are consistent with those reported [2,16].

Higher mean scores in dimensions related to interpersonal relationships suggest that these aspects are perceived more positively by participants, which is in line with findings from [17,18], suggesting that support from colleagues and management can mitigate the negative effects associated with nursing work. This pattern suggests a dichotomy in nurses’ professional experience: on the one hand, satisfaction is derived from relationships with colleagues and from the intrinsic value of their work (particularly recognition from patients); on the other hand, there is significant dissatisfaction with structural aspects of the profession, such as remuneration, career progression, and staffing levels. This polarity between intrinsic and extrinsic aspects of satisfaction is consistent with Herzberg’s Two-Factor Theory [8] and with previous nursing studies [6,10,19,20].

The items with the highest mean scores were related to the intrinsic quality of nursing work (“I am satisfied with the quality of care I provide”, mean = 3.50) and relationships with patients (“I am satisfied with the fact that my work is rewarded and/or valued by patients”, mean = 3.12). In contrast, the lowest mean scores were associated with career progression (“I am satisfied with the time I have to wait for promotion”, mean = 1.45) and remuneration (“I am satisfied with career progression opportunities”, mean = 1.58).

These findings are aligned with previous studies identifying career stagnation and perceived inadequate remuneration as significant sources of dissatisfaction among Portuguese nurses [3,4]. The low level of satisfaction with staffing levels (mean = 2.49) is also noteworthy, given its potential impact on the quality and safety of care. This finding may reflect perceived inadequacies in staffing among participants and is consistent with previous literature highlighting staffing challenges in healthcare settings [20].

Differences in job satisfaction across work schedule groups, consistent with previous findings [2,15], highlight the need to reorganize work practices and strengthen human resource allocation in order to reduce workload burden.

### 4.1. Implications for Nursing Management and Policy

The findings of this study have important implications for nursing management, health policy, and patient safety within the Portuguese healthcare system.

Implications for Nursing Management.

The identification of moderate overall job satisfaction, combined with significant dissatisfaction regarding remuneration, career progression, and staffing levels, highlights the need for targeted and evidence-based managerial interventions. Nursing managers should prioritize the implementation of structured recognition systems, transparent career development pathways, and participatory leadership models that enhance professional autonomy and perceived organizational support.

The significant differences observed according to work setting, working hours, and multiple job holding suggest that workload distribution and staffing adequacy are key determinants of job satisfaction. Investment in safe nurse-to-patient ratios and improved workforce planning may contribute to improved professional well-being and reduced turnover intentions.

Furthermore, strengthening communication between nurses and leadership, promoting continuing professional development, and fostering supportive team environments may enhance intrinsic motivation and organizational commitment.

Implications for Health Policy.

At a broader level, the findings reinforce the urgent need for national workforce policies addressing structural determinants of dissatisfaction, particularly salary conditions, career stagnation, and professional recognition.

Given the high proportion of nurses who reported having considered leaving the profession, workforce retention strategies should be prioritized within national health planning frameworks. Policymakers should consider revising compensation structures, streamlining career progression pathways, and implementing targeted retention incentives, particularly in hospital settings where lower levels of satisfaction were observed.

Investment in the sustainability of the nursing workforce is not only essential for professional well-being but also a strategic priority for healthcare system resilience.

Implications for Patient Safety and Quality of Care.

Job satisfaction is closely associated with care quality, patient safety, and clinical outcomes. Dissatisfaction related to staffing levels and workload may increase the risk of burnout, clinical errors, and compromised care delivery.

Improving working conditions, ensuring adequate staffing, and reinforcing professional recognition may contribute to enhanced patient-centered care, a stronger safety culture, and improved organizational performance.

Therefore, promoting job satisfaction among nurses should be considered a strategic approach to improving quality of care, rather than merely a human resource concern.

### 4.2. Limitations

This study has several limitations that should be considered when interpreting the findings.

First, the use of a non-probabilistic convenience sampling method may introduce selection bias, as participants were recruited based on accessibility and willingness to participate, potentially limiting the representativeness of the sample. Additionally, the relatively low response rate (28.9%) may lead to non-response bias, as those who chose to participate may differ systematically from those who did not. Second, the cross-sectional design precludes the establishment of causal relationships between the variables analyzed, allowing only the identification of statistical associations at a specific point in time. Future longitudinal studies are needed to better understand the directionality and temporal evolution of the relationships between organizational factors, professional conditions, and job satisfaction.

An additional important limitation concerns the exclusive use of univariate analyses. This approach does not allow for the control of potential confounding factors, meaning that the associations identified should be interpreted with caution and cannot be considered independent. Future studies should incorporate multivariate analytical models to provide a more robust understanding of the relationships between variables.

Furthermore, data collection took place in the period following the COVID-19 pandemic, a context that may have influenced professionals’ perceptions of their working conditions, workload, and professional recognition. As such, the levels of satisfaction observed may reflect a specific moment of institutional adjustment and reorganization.

Finally, although several sociodemographic and professional variables were included, other potentially relevant factors were not considered in the analytical model. These include individual characteristics (e.g., personality traits, resilience, or coping strategies), organizational variables (such as leadership styles or organizational climate), and objective indicators of care performance. The inclusion of these dimensions in future research may contribute to a more comprehensive and integrated understanding of job satisfaction in nursing.

## 5. Conclusions

The findings indicate that nurses’ job satisfaction is predominantly moderate and reflects a multidimensional construct. Higher levels of satisfaction were observed in dimensions related to interpersonal relationships and the intrinsic aspects of professional practice, whereas lower levels were identified in areas such as remuneration, career progression, and staffing levels.

Differences in job satisfaction across professional and organizational variables, including work setting, work schedule, workload, and multiple job holding, suggest that contextual factors may contribute to variations in nurses’ experiences. However, given the cross-sectional design, single-center setting, convenience sampling, and reliance on self-reported data, these findings should be interpreted with caution and do not support causal inferences.

The areas of lower satisfaction identified in this study may represent priorities for future organizational assessment and management attention. In this context, aspects such as professional recognition, career development pathways, staffing adequacy, and work schedule organization may be relevant considerations for the design and evaluation of targeted interventions in similar settings.

Additionally, the high proportion of professionals reporting intentions to change departments or having considered leaving the profession highlights potential concerns regarding workforce stability. While these findings do not establish causal relationships, they underscore the importance of further research, particularly using longitudinal and multicenter designs, to better understand the links between organizational factors and professional outcomes.

## 6. Recommendations

Future research should prioritize longitudinal designs to examine changes in job satisfaction over time and to better explore potential relationships between job satisfaction and its associated variables. Expanding studies to include multiple Local Health Units would enable regional comparisons and support the identification of context-specific factors.

The use of mixed-methods approaches is also recommended, combining quantitative and qualitative data to provide a more comprehensive understanding of nurses’ experiences and perceptions. In addition, further research should evaluate the effectiveness of interventions aimed at improving job satisfaction, with a focus on identifying contextually appropriate and evidence-informed strategies.

Exploring the relationship between nurses’ job satisfaction and indicators of care quality and safety may also be valuable, as well as examining the role of potential mediating or moderating factors, such as resilience and emotional intelligence.

In the present study, lower levels of job satisfaction were observed in relation to aspects such as remuneration, career development opportunities, and promotion timelines. These findings suggest that such domains may represent priority areas for organizational attention and future evaluation, rather than reflecting strengths within the current professional context.

## Figures and Tables

**Table 1:** Internal consistency of the dimensions of the ESET scale.

Dimension	Cronbach’s Alpha
Satisfaction with Leadership	0.944
Satisfaction with Organization and Resources	0.905
Satisfaction with Professional Recognition	0.895
Satisfaction with Co-workers	0.916
Satisfaction with Recognition and Remuneration	0.772
Satisfaction with Staffing Levels	0.917

**Table 2:** Sociodemographic and Professional Characterization of the Sample (N = 153).

Variable	Category	n	%
Gender	Female	126	82.4
	Male	25	16.3
	Prefer not to answer	2	1.3
Age	20–30 years	16	10.5
	30–40 years	34	22.2
	40–50 years	52	34.0
	50–60 years	44	28.8
	>60 years	7	4.6
Marital Status	Married	90	58.8
	Single	20	13.1
	Cohabiting	27	17.6
	Divorced/Separated	15	9.8
	Widowed	1	0.7
Children	Yes	117	76.5
	No	36	23.5
Educational Level	Bachelor’s degree	80	52.3
	Postgraduate degree	27	17.6
	Master’s degree	45	29.4
	Other	1	0.7
Nursing Specialty	Yes	68	44.4
	No	85	55.6
Professional Category	Nurse	96	62.7
	Specialist Nurse	47	30.7
	Specialized Nurse	6	3.9
	Nurse Manager	4	2.6
Workplace	Hospital	119	77.8
	Primary Health Care	31	20.3
	Other	3	2.0
Multiple Employment	Yes	38	24.8
	No	115	75.2
Weekly Hours	<35 h	12	7.8
	35–40 h	119	77.8
	>40 h	22	14.4
Years of Professional Experience	1–5 years	10	6.5
	6–10 years	8	5.2
	11–20 years	44	28.8
	21–30 years	50	32.7
	31–40 years	39	25.5
	≥40 years	2	1.3
Work Schedule	Fixed	54	35.3
	Shift work	93	60.8
	Other	6	3.9
Employment Contract	Public service	85	55.6
	Permanent contract	68	44.4

**Table 3:** Distribution of participants according to their intention to change department or leave their current workplace.

Variable	n	%
Intention to change department or leave current workplace		
Yes	46	30.1
No	107	69.9
Considered leaving the nursing profession during career		
Yes	93	60.8
No	60	39.2

**Table 4:** Descriptive statistics of job satisfaction levels.

Job Satisfaction Level	n	%
Low satisfaction	8	5.2
Moderate satisfaction	116	75.8
High satisfaction	29	19.0
Total	153	100.0

**Table 5:** Descriptive statistics (minimum, maximum, mean, and standard deviation) of the ESET items.

ESET Scale Items	Mean	SD	Min	Max
I am satisfied with opportunities for dialogue and sharing information with colleagues.	3.08	0.91	1.00	5.00
I am satisfied with the spirit of collaboration among colleagues.	3.01	1.00	1.00	5.00
I am satisfied with my workload.	2.88	1.01	1.00	5.00
I am satisfied with colleagues’ efforts to provide better care.	2.84	0.99	1.00	5.00
I am satisfied with management efforts to improve working conditions.	2.59	1.12	1.00	5.00
I am satisfied with my participation in decision-making.	2.63	1.05	1.00	5.00
I am satisfied with nurse-to-patient ratios per shift.	2.50	1.16	1.00	5.00
I am satisfied with opportunities for career progression.	1.58	0.82	1.00	4.00
I am satisfied with trust in my colleagues.	2.91	0.93	1.00	5.00
I am satisfied with opportunities provided by management for training/projects.	2.85	1.10	1.00	5.00
I am satisfied with staffing levels related to workload.	2.49	1.05	1.00	5.00
I am satisfied with the physical working conditions.	2.67	0.98	1.00	5.00
I am satisfied with service routines.	2.84	0.93	1.00	5.00
I am satisfied with recognition by management.	2.27	1.10	1.00	5.00
I am satisfied with recognition by patients.	3.12	0.98	1.00	5.00
I am satisfied with recognition from patients and families.	3.07	1.03	1.00	5.00
I am satisfied with working on this service.	3.37	0.94	1.00	5.00
I am satisfied with the competence of other health professionals.	2.82	0.88	1.00	5.00
I am satisfied with training opportunities.	2.75	0.92	1.00	5.00
I am satisfied with colleagues’ professional competence.	3.01	0.87	1.00	5.00
I am satisfied with applying new knowledge in practice.	2.82	0.98	1.00	5.00
I am satisfied with available equipment/materials.	2.80	0.92	1.00	5.00
I am satisfied with workplace organization.	2.76	0.95	1.00	5.00
I am satisfied with autonomy in providing care.	3.35	0.87	1.00	5.00
I am satisfied with protocols in the service.	2.76	1.04	1.00	5.00
I am satisfied with the quality of care I provide.	3.50	0.85	1.00	5.00
I am satisfied with the time required for promotion.	1.45	0.80	1.00	5.00
I am satisfied with my job role.	3.20	0.91	1.00	5.00
I am satisfied with respect from management.	2.83	1.08	1.00	5.00
I am satisfied with the number of protocols in the service.	2.63	1.04	1.00	5.00
I am satisfied with my salary considering my job role.	1.61	0.87	1.00	4.00
I am satisfied with my salary considering my skills/knowledge.	1.59	0.89	1.00	4.00
I am satisfied with communication with management.	2.65	1.07	1.00	5.00
I am satisfied with patients’ perception of my work.	2.87	0.96	1.00	5.00
I am satisfied with management support for training.	2.61	1.00	1.00	5.00
I am satisfied with respect from patients.	2.96	1.01	1.00	5.00
I am satisfied with respect from other health professionals.	2.69	0.93	1.00	4.00

**Table 6:** Descriptive statistics (minimum, maximum, mean, and standard deviation) of the ESET dimensions.

ESET Scale Dimensions	Min	Max	Mean	SD
Satisfaction with Leadership	1.00	5.00	2.83	0.80
Satisfaction with Organization and Resources	1.00	4.88	2.85	0.74
Satisfaction with Professional Recognition	1.00	4.80	2.94	0.82
Satisfaction with Co-workers	1.00	5.00	2.97	0.82
Satisfaction with Recognition and Remuneration	1.00	3.60	1.82	0.64
Satisfaction with Staffing Levels	1.00	5.00	2.49	1.06

**Table 7:** Significance of differences in ESET dimensions across sociodemographic and professional variables.

Variables	Category	1 M (SD)	2 M(SD)	3 M (SD)	4 M(SD)	5 M (SD)	6 M(SD)
Gender (Independent samples *t*-test)	Female	2.85 (0.81)	2.86 (0.71)	2.91 (0.83)	2.94 (0.79)	1.85 (0.64)	2.49 (1.04)
	Male	2.75 (0.73)	2.87 (0.84)	3.12 (0.80)	3.14 (0.94)	1.67 (0.57)	2.56 (1.19)
	*p*-value	0.281	0.463	0.123	0.128	0.101	0.379
	Cohen’s d	0.127	−0.020	−0.255	−0.250	0.281	−0.068
Age (One-way ANOVA)	20–30 years	2.55 (0.80)	2.77 (0.82)	2.86 (0.82)	3.00 (0.90)	1.75 (0.59)	2.44 (1.33)
	30–40 years	2.78 (0.84)	2.77 (0.73)	2.67 (0.88)	2.92 (0.84)	1.67 (0.58)	2.50 (1.00)
	40–50 years	2.91 (0.90)	2.83 (0.89)	3.07 (0.85)	2.99 (0.92)	1.91 (0.66)	2.52 (1.18)
	50–60 years	2.76 (0.61)	2.87 (0.52)	2.97 (0.73)	2.90 (0.65)	1.84 (0.65)	2.45 (0.95)
	>60 years	3.45 (0.65)	3.41 (0.26)	3.31 (0.66)	3.43 (0.68)	1.97 (0.78)	2.64 (0.63)
	*p*-value	0.132	0.323	0.153	0.600	0.486	0.992
	η^2^	0.046	0.046	0.044	0.018	0.023	0.002
Marital Status (Welch’s ANOVA)	Single	2.83 (0.80)	2.84 (0.70)	2.83 (0.87)	3.42 (0.63)	1.77 (0.61)	2.48 (1.19)
	Married	2.94 (0.80)	2.95 (0.74)	3.05 (0.74)	2.98 (0.82)	1.96 (0.64)	2.61 (1.04)
	Cohabiting	2.60 (0.78)	2.71 (0.73)	2.83 (0.95)	2.81 (0.85)	1.59 (0.58)	2.20 (1.03)
	Divorced/Separated	2.59 (0.81)	2.54 (0.73)	2.79 (0.89)	2.64 (0.73)	1.56 (0.58)	2.37 (1.09)
	*p*-value	0.157	0.161	0.401	0.022 *	0.014 *	0.344
	η^2^	0.035	0.034	0.020	0.063	0.069	0.022
Children (Independent samples *t*-test)	Yes	2.82 (0.81)	2.84 (0.76)	3.00 (0.81)	2.92 (0.84)	1.82 (0.63)	2.49 (1.05)
	No	2.84 (0.77)	2.87 (0.67)	2.77 (0.86)	3.13 (0.73)	1.84 (0.66)	2.50 (1.11)
	*p*-value	0.435	0.420	0.070	0.083	0.429	0.483
	Cohen’s d	−0.025	−0.033	0.306	−0.259	−0.023	−0.004
Education Level (One-way ANOVA)	Bachelor’s degree	2.85 (0.77)	2.89 (0.73)	2.90 (0.84)	2.96 (0.76)	1.87 (0.71)	2.40 (1.05)
	Postgraduate	2.59 (0.99)	2.56 (0.92)	2.93 (0.75)	2.77 (1.00)	1.59 (0.46)	2.63 (1.17)
	Master’s degree	2.91 (0.71)	2.94 (0.59)	3.03 (0.86)	3.09 (0.78)	1.87 (0.59)	2.59 (1.02)
	*p*-value	0.235	0.078	0.701	0.269	0.126	0.494
	η^2^	0.019	0.034	0.005	0.017	0.027	0.009
Specialization in Nursing (Independent samples *t*-test)	Yes	2.88 (0.81)	2.88 (0.69)	3.05 (0.79)	2.97 (0.82)	1.84 (0.65)	2.59 (1.08)
	No	2.78 (0.80)	2.83 (0.77)	2.86 (0.84)	2.96 (0.81)	1.80 (0.63)	2.42 (1.05)
	*p*-value	0.213	0.352	0.071	0.474	0.353	0.162
	Cohen’s d	0.130	0.062	0.240	0.011	0.062	0.161
Work Setting (Independent samples *t*-test)	Hospital	2.78 (0.78)	2.87 (0.75)	2.86 (0.86)	2.95 (0.83)	1.74 (0.59)	2.41 (1.07)
	Primary healthcare	2.88 (0.83)	2.71 (0.66)	3.20 (0.56)	3.03 (0.79)	2.17 (0.72)	2.79 (1.04)
	*p*-value	0.283	0.146	0.006 ***	0.320	0.002 ***	0.039 *
	Cohen’s d	−0.116	0.213	−0.413	−0.094	−0.691	−0.357
Multiple Workplaces (Independent samples *t*-test)	Yes	2.62 (0.80)	2.64 (0.79)	2.81 (0.84)	2.89 (0.76)	1.55 (0.47)	2.16 (1.10)
	No	2.89 (0.79)	2.92 (0.71)	2.99 (0.81)	2.99 (0.83)	1.91 (0.66)	2.60 (1.03)
	*p*-value	0.033 *	0.020 *	0.125	0.246	0.001 ***	0.016 *
	Cohen’s d	−0.348	−0.386	−0.216	−0.129	−0.577	−0.427
Weekly Working Hours (One-way ANOVA)	<35 h	3.04 (0.92)	2.90 (0.87)	3.32 (0.93)	2.98 (0.78)	1.83 (0.67)	2.67 (1.42)
	35–40 h	2.82 (0.81)	2.85 (0.74)	2.91 (0.80)	2.97 (0.84)	1.88 (0.66)	2.53 (1.00)
	>40 h	2.75 (0.68)	2.82 (0.64)	2.91 (0.87)	2.94 (0.69)	1.53 (0.37)	2.23 (1.16)
	*p*-value	0.607	0.958	0.264	0.979	0.007 ***	0.407
	η^2^	0.007	0.001	0.018	0.00	0.036	0.012
Years of Experience (One-way ANOVA)	1–5 years	2.83 (0.75)	3.00 (0.91)	3.16 (0.78)	3.44 (0.57)	1.82 (0.64)	2.70 (1.42)
	6–10 years	2.21 (0.68)	2.47 (0.49)	2.33 (0.71)	2.45 (0.83)	1.78 (0.67)	2.13 (1.03)
	11–20 years	2.83 (0.89)	2.82 (0.80)	2.85 (0.96)	2.86 (0.86)	1.66 (0.57)	2.44 (1.08)
	21–30 years	2.92 (0.82)	2.85 (0.80)	3.00 (0.78)	3.04 (0.89)	1.95 (0.69)	2.58 (1.11)
	31–40 years	2.78 (0.66)	2.88 (0.56)	3.00 (0.68)	2.97 (0.63)	1.86 (0.62)	2.46 (0.91)
	*p*-value	0.237	0.617	0.188	0.100	0.308	0.778
	η^2^	0.037	0.018	0.041	0.051	0.032	0.012
Work Schedule (Independent samples *t*-test)	Fixed	3.12 (0.81)	3.01 (0.69)	3.20 (0.79)	3.05 (0.84)	2.09 (0.65)	2.80 (0.99)
	Shift work	2.68 (0.73)	2.77 (0.72)	2.76 (0.81)	2.95 (0.77)	1.68 (0.56)	2.39 (1.06)
	*p*-value	0.001 ***	0.028 *	0.001 ***	0.255	0.001 ***	0.034 *
	Cohen’s d	0.609	0.367	0.580	0.117	0.664	0.362
Employment Contract (Independent samples *t*-test)	Public contract	2.79 (0.75)	2.82 (0.69)	2.96 (0.76)	2.98 (0.76)	1.88 (0.67)	2.49 (1.00)
	Permanent contract	2.85 (0.86)	2.86 (0.80)	2.89 (0.88)	2.95 (0.88)	1.76 (0.59)	2.49 (1.15)
	*p*-value	0.334	0.356	0.308	0.425	0.118	0.497
	Cohen’s d	−0.045	−0.035	0.109	−0.284	−0.133	−0.318

Note: (1) Satisfaction with leadership; (2) Satisfaction with organization and resources; (3) Satisfaction with professional recognition; (4) Satisfaction with co-workers; (5) Satisfaction with recognition and remuneration; (6) Satisfaction with staffing levels; M—Mean; SD—Standard deviation; * *p* ≤ 0.05; *** *p* ≤ 0.001. Independent samples *t*-tests were used for dichotomous variables when assumptions were met; Welch’s *t*-test was applied when homogeneity of variances was violated. One-way ANOVA was used for comparisons involving three or more groups, with Welch ANOVA applied when variance homogeneity was not met. Non-parametric alternatives (Mann–Whitney U or Kruskal–Wallis tests) were used when normality assumptions were violated. Effect sizes are reported as Cohen’s d for *t*-tests and η^2^ for ANOVA.

## Data Availability

Due to ethical issues, the data collected and analysed in this study are not available to outside researchers.

## Public Involvement Statement

Patients and members of the public were not involved in the design, conduct, reporting, or dissemination plans of this research. The study focused exclusively on nurses’ perceptions of job satisfaction within a healthcare organizational context.

## Guidelines and Standards Statement

This manuscript was drafted against the STROBE (Strengthening the Reporting of Observational Studies in Epidemiology) Statement for cross-sectional observational research. The authors ensured that the study was reported transparently and consistently with the methodological approach adopted.

## Abbreviations

The following abbreviations are used in this manuscript:

ESET	Escala de Satisfação dos Enfermeiros com o Trabalho
LHUCB	Local Health Unit of Castelo Branco
SPSS	Statistical Package for the Social Sciences
LSD	Least Significant Difference
